# Dose optimization approach to fast X-ray microtomography of the lung alveoli

**DOI:** 10.1107/S0021889813005591

**Published:** 2013-06-07

**Authors:** Goran Lovric, Sébastien F. Barré, Johannes C. Schittny, Matthias Roth-Kleiner, Marco Stampanoni, Rajmund Mokso

**Affiliations:** a Swiss Light Source, Paul Scherrer Institute, 5234 Villigen, Switzerland; b Institute for Biomedical Engineering, University and ETH Zurich, 8092 Zurich, Switzerland; c Institute of Anatomy, University of Bern, 3012 Bern, Switzerland; d Graduate School for Cellular and Biomedical Sciences, University of Bern, 3012 Bern, Switzerland; e Clinic of Neonatology, University Hospital of Lausanne (CHUV), 1011 Lausanne, Switzerland

**Keywords:** dose optimization, radiation dose, X-ray microtomography, lung aveoli

## Abstract

A basic prerequisite for *in vivo* X-ray imaging of the lung is the exact determination of radiation dose. Achieving resolutions of the order of micrometres may become particularly challenging owing to increased dose, which in the worst case can be lethal for the imaged animal model. A framework for linking image quality to radiation dose in order to optimize experimental parameters with respect to dose reduction is presented. The approach may find application for current and future *in vivo* studies to facilitate proper experiment planning and radiation risk assessment on the one hand and exploit imaging capabilities on the other.

## Abstract 2

A framework for linking image quality to radiation dose in order to optimize experimental parameters with respect to dose reduction is presented.

## 1. Introduction

The development of high-speed time-resolved tomographic microscopy is of great interest for various three-dimensional *in vivo* studies. One important application is the study of lung dynamics, in particular lung inflation and deflation issues during physiological as well as mechanical ventilation, which is required, for example, after a premature birth or during a general anesthetic. Two hypotheses on the structural alterations in the gas-exchange area during breathing are still under debate: a heterogeneous distention pattern (Mertens *et al.*, 2009 ▶) of different lung areas and a homogeneous cyclic opening-and-collapse (Albert *et al.*, 2009 ▶) of all alveoli.

In the recent past, lung imaging with small animal models has become an established technique at synchrotrons (Yagi *et al.*, 1999 ▶; Bayat *et al.*, 2001 ▶; Lewis *et al.*, 2005 ▶), making rapid data acquisition and high-resolution X-ray imaging possible (Flannery *et al.*, 1987 ▶). Kitchen *et al.* (2004 ▶) performed phase-contrast X-ray imaging of mice and rabbits *in vivo* in two-dimensions and developed a method for visualizing lung liquid clearance at birth (Kitchen *et al.*, 2005 ▶; Hooper *et al.*, 2009 ▶). Bayat *et al.* (2001 ▶) used xenon as a contrast agent and were able to measure and visualize gas distribution within the lungs in three dimensions, enabling further functional studies (Suhonen *et al.*, 2008 ▶; Bayat *et al.*, 2008 ▶, 2009 ▶). More recently, synchrotron-based tomographic microscopy has been utilized to study the distribution of gas flow throughout the airway tree in connection with altered lung motion as an indicator for regional lung disease (Fouras *et al.*, 2012 ▶; Dubsky *et al.*, 2012 ▶).

So far, all studies either were performed in two dimensions only or suffered from low temporal and spatial resolution; high-resolution lung images in three dimensions, on the other hand, were successfully obtained only for static samples (Schittny *et al.*, 2008 ▶; Yong *et al.*, 2009 ▶; Haberthür *et al.*, 2010 ▶; Mokso *et al.*, 2011 ▶; Zhang *et al.*, 2011 ▶). Thus, ***in vivo*** X-ray tomography with micrometre spatial and sub-second temporal resolution remains a challenge and many open questions in the study of lung physiology remain unanswered.

We describe our approach to image formation of biologically relevant features in the lung, aiming at optimal image quality in terms of contrast, spatial and temporal resolution, and deposited radiation dose. In particular, we show current limitations towards **in vivo** imaging at the micrometre scale.

## 2. Materials and methods

### 2.1. Image acquisition

**Figure 1:**
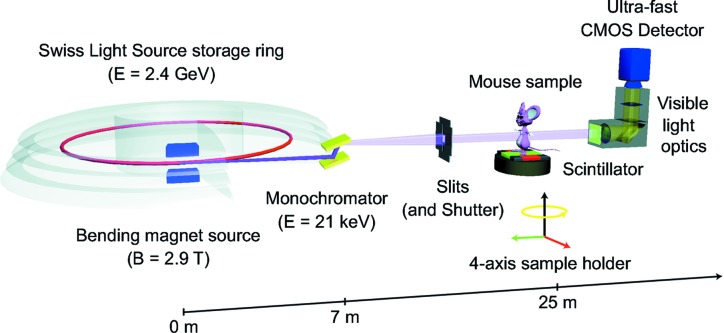
Experimental setup at the X02DA TOMCAT beamline.

The experiment was carried out at the X02DA TOMCAT beamline of the Swiss Light Source (SLS) at the Paul Scherrer Institute (Villigen, Switzerland). The experimental setup is depicted in Fig. 1 ▶: the X-ray beam, produced by a 2.9 T bending magnet on a 2.4 GeV storage ring (with ring current *I* = 400 mA, top-up mode), is monochromated with a double-multilayer monochromator and tuned to 21 keV.

A sample-to-source distance of 25 m is used for producing an X-ray beam with appropriate spatial coherence properties. We used a high-speed CMOS detector (PCO.Dimax) coupled to visible-light optics with 100 and 20 µm-thick scintillators for medium and high spatial resolutions, respectively. The samples were probed with two different optics, yielding effective pixel sizes of 2.9 and 1.1 µm, respectively. For these two optics the field of view was adjusted with horizontal and vertical slits, located just before the sample and producing beam sizes of 5.8 × 2.7 mm and 2.2 × 2.2 mm, respectively. The sample-to-detector distance *z* was varied in the range 24–300 mm to allow for the variation of the Fresnel interference pattern for image quality optimization purposes. Raw images were acquired with exposure times ranging from 2 to 13 ms and 901 tomographic projections.

The measurements were performed *ex vivo* on mice aged 37 d (*n* = 3 / Balb-C, central animal facility of the University of Bern) that were sacrificed before the experiment. The mice were killed with an overdose of a combination of Acepromazine, Xylazin and Ketamin. They were then placed in an upright position into a 2.5 cm-diameter Falcon tube. All parts of the animal experiments were approved and supervised by the Swiss Agency for the Environment, Forest and Landscape, and the Veterinary Service of the Canton of Bern.

### 2.2. Post-processing

**Figure 2:**
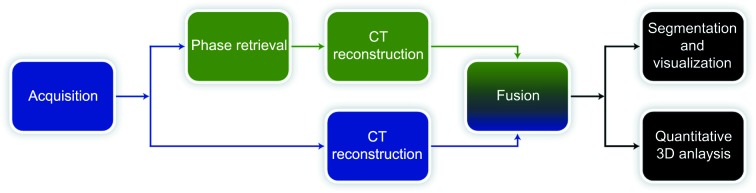
Flowchart of the post-processing pipeline. After image acquisition, the CT-reconstructed volumes obtained from absorption and phase-retrieved images are fused in the Fourier domain. Afterwards, further analysis (segmentation, visualization *etc*.) can be applied.

The aforementioned setup facilitates propagation-based phase contrast, represented by interference fringes on the tissue interfaces in the recorded digital projection images. Prior to further analysis, each projection was corrected with the respective dark and flat-field image. In a second step, single-image phase and intensity extraction were applied to all projections (Paganin *et al.*, 2002 ▶). In a third step, computed tomography (CT) reconstruction was conducted with the *gridrec* algorithm (Marone & Stampanoni, 2012 ▶), enabling fast reconstructions of large data sets. Finally, the CT reconstructions obtained from phase-retrieved images and absorption images were fused in the Fourier domain in order to correct for the high-pass characteristics of the Paganin algorithm (Irvine *et al.*, 2013 ▶). The complete post-processing flowchart is depicted in Fig. 2 ▶.

### 2.3. Image analysis

The reconstructed tomographic slices were examined in view of contrast-to-noise ratio (CNR) and resolution. For CNR the following formula was used:
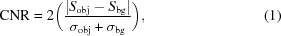
where *S_i_* represent mean pixel values and σ_*i*_ standard deviations of a manually defined object and background region, respectively. One has to consider, however, that the object (*i.e.* lung tissue) consists of blood vessels and erythrocytes which have different densities and may lead to distorted contrast levels. Under the present imaging scheme, this effect is negligible.

The resolution was determined by a criterion based upon Fourier analysis (Modregger *et al.*, 2007 ▶). Considering a test image the resolution was obtained by taking a line profile and calculating its power spectral density (PSD). The PSD converges towards a value that can be defined as the noise baseline. Taking the value of the PSD at the noise baseline twice and matching it to the respective spatial frequency yields the resolution. This can be made more robust and fully automated if one operates on the mean PSD, obtained from many line profiles. The only necessary condition for this method requires the highest spatial frequency of the image to be lower than the Nyquist frequency of the line profile. Since the smallest features of the lung are at least of the order of tens of pixels, this condition was fulfilled for both optics of our setup.

Additionally, the results were crosschecked with another method by which the resolution is derived from a line profile taken along an edge in the image. The line profile is then fitted with an error function by means of a least-squares fit and the resolution is determined from the slope of the function.

**Figure 3:**
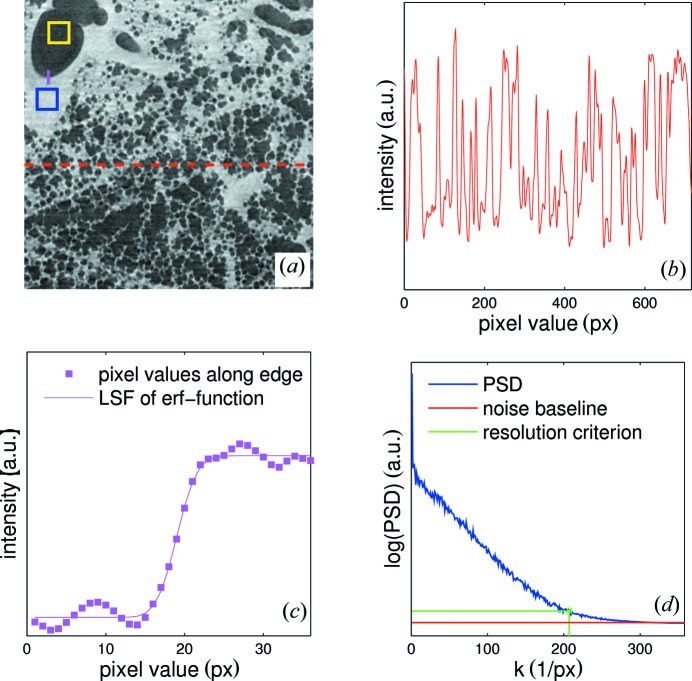
(*a*) A tomographic slice of a lung for the 2.9 µm-pixel-size optics. Regions for calculating CNR are defined by the rectangles, the red dashed line indicates the input for the line-profile plot in (*b*) and the solid magenta line indicates the edge line plot in (*c*). (*d*) Mean PSD from many line plots with the calculated noise baseline and the respective resolution criterion.

In Fig. 3 ▶ the test image with the defined object and background regions, the line profile, the line profile along the edge, and the calculated PSD are shown. The image in Fig. 3 ▶(*a*) was constructed from a region near the center of the original tomographic slice in order to reduce the radial dependency of the resolution originating from the tomographic reconstruction. The resolution *x*_res_ is calculated by
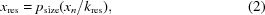
where *p*_size_ is the pixel size of the detector, *x_n_* the number of pixels for the taken line profile and *k*_res_ the spatial frequency obtained from the resolution criterion in Fig. 3 ▶(*d*).

### 2.4. Dose calculations

The X-ray radiation dose was assessed in two steps by theoretical and experimental means. First, the theoretical flux 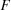 was calculated with the TOMCAT beamline parameters (Stampanoni *et al.*, 2006 ▶) and the following formula (Kim, 1995 ▶):
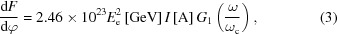
with electron energy *E*_e_, ring current *I* and 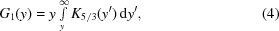
where ω_c_ is the critical frequency of the bending-magnet radiation, ω is the emitted photon frequency and *K*_5/3_ represents the modified Bessel function of the second kind. In equation (3) the flux is given in practical units [photons s^−1^ mrad^−1^ (0.1%, bandwidth)^−1^]. Thus it has to be further corrected in order to include bandwidth and reflectivity of the monchromator as well as all optical elements that further reduce the theoretical flux. In a second step, the absorbed dose (Asadchikov *et al.*, 2010 ▶) of a 2.5 cm water column was calculated from NIST mass attenuation coefficients (http://physics.nist.gov/PhysRefData/XrayMassCoef/ComTab/water.html).

**Figure 4:**
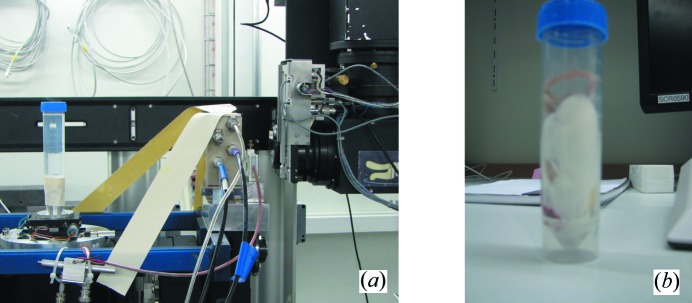
Dose and flux measuring setup. (*a*) Water column with reference ionization chamber and a silicon pin diode (placed behind the ionization chamber. (*b*) Frozen mouse sample.

The X-ray flux was determined experimentally with a calibrated silicon pin diode (Owen *et al.*, 2009 ▶) and a frozen mouse sample in connection with a 2.5 cm water column to verify that the modeling with water is appropriate. Images from the setup are depicted in Fig. 4 ▶.

## 3. Results

**Figure 5:**
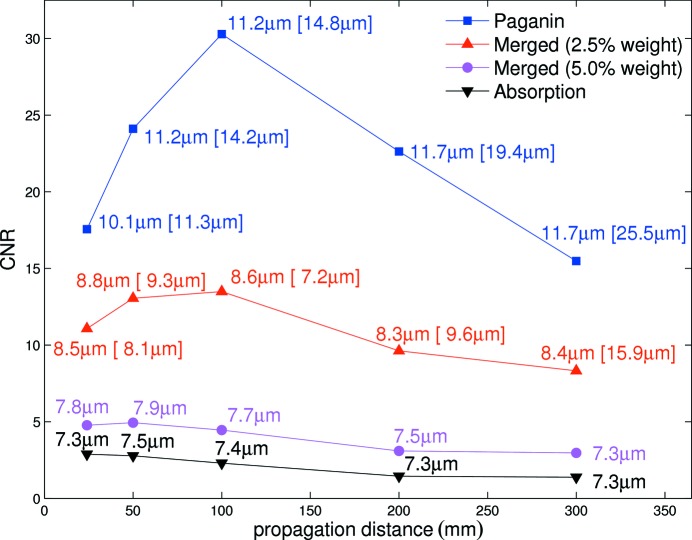
Contrast-to-noise ratio (CNR) as a function of sample-to-detector (propagation) distance and the weighting of the absorption image in the fusion algorithm. The values were calculated for the 2.9 µm-pixel-size optics and 11.7 s total scan time. The values in brackets denote resolutions obtained from the edge-fitting method.

Lung images were reconstructed from tomographic projections taken at five different propagation distances. Fig. 5 ▶ shows the calculated CNR and resolution for each distance and for different weighting factors for the absorption images in the fusion algorithm. The values in brackets denote resolutions obtained from the edge-fitting method. As expected, for the transport-of-intensity-based phase-retrieval approach in the near-field region the optimal propagation distance is near the value of 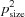/λ (m), where *p*_size_ is the pixel size and λ is the wavelength. We show that fine tuning this distance can significantly affect CNR. By applying the fusion algorithm (Irvine *et al.*, 2013 ▶), it is possible to increase the resolution further, but at a cost of CNR. This can be used for adjusting the resolution of the reconstructed image.

**Figure 6:**
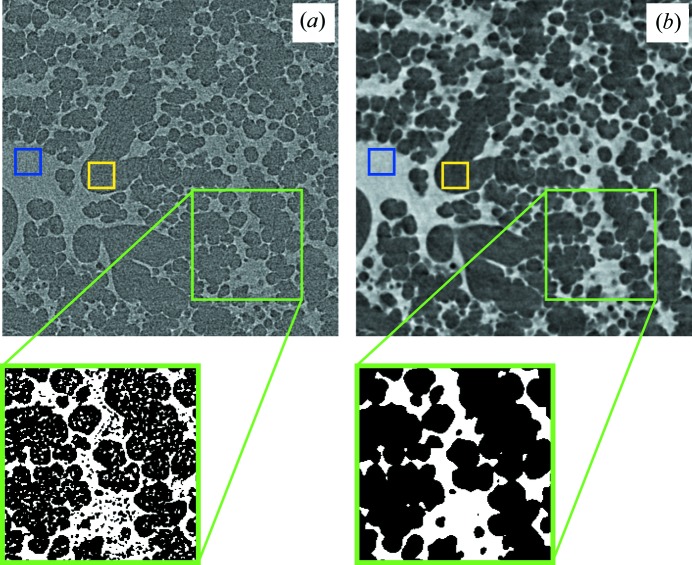
Comparison of tomographic slices before and after thresholding obtained from pure absorption-contrast (*a*) and phase-retrieved projection images (*b*). For both images the total scan time was 5.4 s. The small rectangles denote the regions for calculating CNR.

It has been reported elsewhere (Lewis, 2004 ▶; Sera *et al.*, 2008 ▶) that phase-contrast images of lungs exhibit better contrast and especially less noise than pure absorption images. We addressed this issue by evaluating the degree of ‘segmentability’ in a tomographic scan. In Fig. 6 ▶, two images and the respective binary images obtained by thresholding are plotted. The total scan time was 5.4 s and the CNR values for the absorption and phase-retrieved images were 1.8 and 15.0, respectively, which makes up a difference of approximately one order of magnitude. However, the segmentation from the absorption images shows small artefacts originating from the higher noise level. We further found that a CNR of ∼2 is currently the lower limit for a successful segmentation and in some cases necessitates further processing (*e.g.* filtering). This limit was verified by visual inspection.

**Figure 7:**
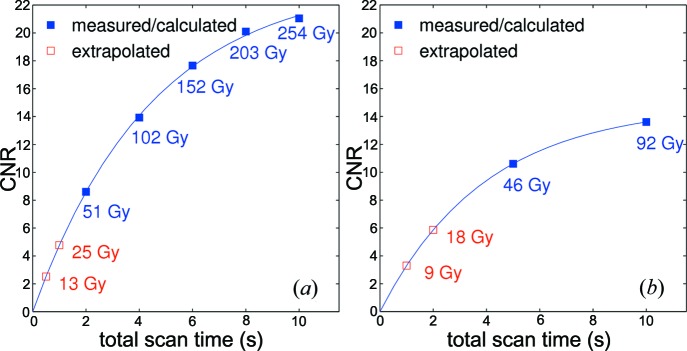
CNR as a function of total scan time per tomographic scan for the two different optics in use. (*a*) Optics with 2.9 µm effective pixel size. (*b*) Optics with 1.1 µm pixel size.

Finally, CNR and the X-ray radiation dose were calculated as functions of the total scan time of a tomographic scan, as shown in Fig. 7 ▶. The values for CNR obtained from experimental data were fitted and extrapolated using the function
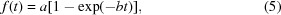
where *a, b* represent arbitrary parameters for the nonlinear fit and *t* the total scan time. The function derives from the fact that CNR will be zero if the sample is not exposed to X-rays (*t* = 0). Thus its origin is at zero. The upper value of CNR is limited by the mean pixel values represented by the density values of the biological material and their standard deviations, which are inherent to the acquisition scheme. CNR will therefore converge to a saturation value in the tomographic reconstruction.

## 4. Discussion

The optimization of CNR with respect to the deposited radiation dose is crucial in low-dose experiments. In particular, we found that the application of the one-shot phase-retrieval algorithm (Paganin *et al.*, 2002 ▶) increased CNR by almost a factor of ten compared with the pure absorption-contrast images. However the spatial resolution decreased, as shown in Fig. 5 ▶. It is also evident that tuning the propagation distance can affect CNR by a factor of two under the given conditions. These results indicate that the optimal propagation distance, represented by a trade-off between CNR and resolution, and fusion parameters are particularly important and should be considered before the experiment. Furthermore, we found that the resolutions obtained from the two different criteria are not consistent for larger propagation distances and the robustness of the edge-fitting method in general was bad. This can be explained as follows: by increasing the propagation distance the edge enhancement becomes more pronounced and the Fourier resolution criterion detects edge artefacts as ‘features’, whereas the edge fitting gives a poorer result. Thus, the Fourier resolution criterion has to be handled with care for larger propagation distances.

For the radiation dose we found that the 1.1 µm-pixel optics (numerical aperture: 0.4) yielded a lower dose than the 2.9 µm-pixel optics (numerical aperture: 0.2) for the same CNR values. The main reason for this result is the fact that images for the two optics were not acquired under the same experimental conditions, *i.e.* optimized propagation distance *etc*. Apart from that, one also has to regard the efficiencies of the different optics. Finally, another reason is the fact that images from both optics were reconstructed from local tomographic projections. Since we calculated the total dose in the irradiated volume, the smaller field-of-view means lower energy deposition for the same volume. Obviously, the dose rate in the lungs will be higher for the 1.1 µm-pixel optics.

The model function from equation (5) for extrapolating CNR as a function of the total scan time still needs to be verified with the *gridrec* algorithm (Marone & Stampanoni, 2012 ▶). However, taking the range of interest that we analyzed, a similar fit (*e.g.* linear) would only cause negligible errors. From the results in Figs. 6 ▶ and 7 ▶ we further hypothesize that under the given conditions the lowest achievable dose for obtaining the necessary CNR is 10 and 5 Gy, respectively.

## 5. Conclusion

We have developed a framework for optimizing experimental parameters in medium-dose experiments, which is particularly important for current efforts in developing microtomographic ***in vivo*** X-ray imaging to study lung physiology at the micrometre scale. Thanks to the fast phase-retrieval and CT reconstruction algorithms, it is possible to apply the optimization steps on-the-fly (or even in real time) as an initial part of a beamline experiment.

We showed that the lowest achievable dose at the moment is in the range of 5–10 Gy per tomographic scan at a total scan time of approximately 0.5 s and approximate resolutions between 4–10 µm, producing images with an approximate CNR of 2. Our results indicate that ***in vivo*** tomography at the micrometre scale and sub-second temporal resolution should be feasible, but will necessitate further adjustments.

## References

[bb1] Albert, S. P., DiRocco, J., Allen, G. B., Bates, J. H., Lafollette, R., Kubiak, B. D., Fischer, J., Maroney, S. & Nieman, G. F. (2009). *J. Appl. Physiol.* **106**, 757–765. 10.1152/japplphysiol.90735.2008 PMC2660249 19074576

[bb2] Asadchikov, V. E., Buzmakov, A. V., Zolotov, D. A., Senin, R. A. & Geranin, A. S. (2010). *Crystallogr. Rep.* **55**, 158–167.

[bb3] Bayat, S., Le Duc, G., Porra, L., Berruyer, G., Nemoz, C., Monfraix, S., Fiedler, S., Thomlinson, W., Suortti, P., Standertskjöld-Nordenstam, C. G. & Sovijärvi, A. R. (2001). *Phys. Med. Biol.* **46**, 3287–3299. 10.1088/0031-9155/46/12/315 11768506

[bb4] Bayat, S., Porra, L., Suhonen, H., Janosi, T., Strengell, S., Habre, W., Petak, F., Hantos, Z., Suortti, P. & Sovijärvi, A. (2008). *Eur. J. Radiol.* **68**, S78–83. 10.1016/j.ejrad.2008.04.043 18606518

[bb5] Bayat, S., Porra, L., Suhonen, H., Suortti, P. & Sovijärvi, A. R. (2009). *J. Appl. Physiol.* **106**, 1949–1958. 10.1152/japplphysiol.90550.2008 19359611

[bb6] Dubsky, S., Hooper, S. B., Siu, K. K. W. & Fouras, A. (2012). *J. R. Soc. Interface*, **9**, 2213–2224. 10.1098/rsif.2012.0116 PMC3405755 22491972

[bb7] Flannery, B. P., Deckman, H. W., Roberge, W. G. & D’Amico, K. L. (1987). *Science*, **237**, 1439–1444. 10.1126/science.237.4821.1439 17816787

[bb8] Fouras, A., Allison, B. J., Kitchen, M. J., Dubsky, S., Nguyen, J., Hourigan, K., Siu, K. K. W., Lewis, R. A., Wallace, M. J. & Hooper, S. B. (2012). *Ann. Biomed. Eng.* **40**, 1160–1169. 10.1007/s10439-011-0493-0 22189492

[bb9] Haberthür, D., Hintermüller, C., Marone, F., Schittny, J. C. & Stampanoni, M. (2010). *J. Synchrotron Rad.* **17**, 590–599. 10.1107/S0909049510019618 PMC2927902 20724780

[bb10] Hooper, S. B., Kitchen, M. J., Siew, M. L., Lewis, R. A., Fouras, A., te Pas, A. B., Siu, K. K. W., Yagi, N., Uesugi, K. & Wallace, M. J. (2009). *Clin. Exp. Pharmacol. Physiol.* **36**, 117–125. 10.1111/j.1440-1681.2008.05109.x 19205087

[bb11] Irvine, S. C., Mokso, R., Modregger, P. *et al.* (2013). In preparation.

[bb12] Kim, K. J. (1995). *Opt. Eng.* **34**, 342.

[bb13] Kitchen, M. J., Lewis, R. A., Yagi, N., Uesugi, K., Paganin, D., Hooper, S. B., Adams, G., Jureczek, S., Singh, J., Christensen, C. R., Hufton, A. P., Hall, C. J., Cheung, K. C. & Pavlov, K. M. (2005). *Br. J. Radiol.* **78**, 1018–1027. 10.1259/bjr/13024611 16249603

[bb14] Kitchen, M. J., Paganin, D., Lewis, R. A., Yagi, N., Uesugi, K. & Mudie, S. T. (2004). *Phys. Med. Biol.* **49**, 4335–4348. 10.1088/0031-9155/49/18/010 15509069

[bb15] Lewis, R. A. (2004). *Phys. Med. Biol.* **49**, 3573–3583. 10.1088/0031-9155/49/16/005 15446788

[bb16] Lewis, R. A., Yagi, N., Kitchen, M. J., Morgan, M. J., Paganin, D., Siu, K. K. W., Pavlov, K., Williams, I., Uesugi, K., Wallace, M. J., Hall, C. J., Whitley, J. & Hooper, S. B. (2005). *Phys. Med. Biol.* **50**, 5031–5040. 10.1088/0031-9155/50/21/006 16237239

[bb17] Marone, F. & Stampanoni, M. (2012). *J. Synchrotron Rad.* **19**, 1029-1037. 10.1107/S0909049512032864 PMC3480277 23093766

[bb18] Mertens, M., Tabuchi, A., Meissner, S., Krueger, A., Schirrmann, K., Kertzscher, U., Pries, A. R., Slutsky, A. S., Koch, E. & Kuebler, W. M. (2009). *Crit. Care Med.* **37**, 2604–2611. 10.1097/CCM.0b013e3181a5544d 19623041

[bb19] Modregger, P., Lübbert, D., Schäfer, P. & Köhler, R. (2007). *Phys. Status Solidi A*, **204**, 2746–2752.

[bb20] Mokso, R., Marone, F., Haberthür, D., Schittny, J. C., Mikuljan, G., Isenegger, A. & Stampanoni, M. (2011). *The 10th International Conference on X-ray Microscopy*, AIP Conference Proceedings Vol. 1365, pp. 38–41. Melville: American Institute of Physics.

[bb21] Owen, R. L., Holton, J. M., Schulze-Briese, C. & Garman, E. F. (2009). *J. Synchrotron Rad.* **16**, 143–151. 10.1107/S0909049508040429 PMC2651761 19240326

[bb22] Paganin, D., Mayo, S. C., Gureyev, T. E., Miller, P. R. & Wilkins, S. W. (2002). *J. Microsc.* **206**, 33–40. 10.1046/j.1365-2818.2002.01010.x 12000561

[bb23] Schittny, J. C., Mund, S. I. & Stampanoni, M. (2008). *Am. J. Physiol. Lung Cell. Mol. Physiol.* **294**, L246–L254. 10.1152/ajplung.00296.2007 18032698

[bb24] Sera, T., Yokota, H., Fujisaki, K., Fukasaku, K., Tachibana, H., Uesugi, K., Yagi, N. & Himeno, R. (2008). *Phys. Med. Biol.* **53**, 4285–4301. 10.1088/0031-9155/53/16/005 18653926

[bb25] Stampanoni, M., Groso, A., Isenegger, A., Mikuljan, G., Chen, Q., Bertrand, A., Henein, S., Betemps, R., Frommherz, U., Böhler, P., Meister, D., Lange, M. & Abela, R. (2006). *Proc. SPIE*, **6318**, 63180M.

[bb26] Suhonen, H., Porra, L., Bayat, S., Sovijärvi, A. R. A. & Suortti, P. (2008). *Phys. Med. Biol.* **53**, 775–791. 10.1088/0031-9155/53/3/016 18199914

[bb27] Yagi, N., Suzuki, Y., Umetani, K., Kohmura, Y. & Yamasaki, K. (1999). *Med. Phys.* **26**, 2190–2193. 10.1118/1.598735 10535637

[bb28] Yong, H. S., Kang, E. Y., Kim, Y. K., Woo, O. H., Shin, B. K., Oh, C. H., Je, J. H., Han, H. & Seo, J. S. (2009). *Yonsei Med. J.* **50**, 422–426. 10.3349/ymj.2009.50.3.422 PMC2703767 19568606

[bb29] Zhang, L., Li, D. & Luo, S. (2011). *PloS ONE*, **6**, e17400. 10.1371/journal.pone.0017400 PMC3045447 21364899

